# Radiation-Induced Lumbosacral Radiculopathy: A Comprehensive Clinical and Dosimetric Study

**DOI:** 10.7759/cureus.100356

**Published:** 2025-12-29

**Authors:** Zineb Dahbi, Reyzane El Mjabber, Amal Bouziyane, Nawal Bouknani, Wissam Bezzari, Rim Alami, Vincent Vinh-Hung, Aboubacar H Bambara

**Affiliations:** 1 Radiation Oncology, Mohammed VI University of Sciences and Health, Casablanca, MAR; 2 Radiation Oncology, Cheikh Khalifa International University Hospital, Casablanca, MAR; 3 Obstetrics and Gynecology, Mohammed VI University of Sciences and Health, Casablanca, MAR; 4 Obstetrics and Gynecology, Cheikh Khalifa International University Hospital, Casablanca, MAR; 5 Radiology, Mohammed VI University of Sciences and Health, Casablanca, MAR; 6 Radiology, Cheikh Khalifa International University Hospital, Casablanca, MAR; 7 Radiation Oncology, Centre Hospitalier Public Du Cotentin, Cherbourg-En-Cotentin, FRA; 8 Medical Oncology, Teaching Hospital Yalgado Ouédraogo, Ouagadougou, BFA

**Keywords:** dosimetric analysis, lumbosacral plexopathy, pelvic radiotherapy, quality of life, radiation toxicity

## Abstract

Background: Radiation-induced lumbosacral radiculopathy is an under-reported late complication of pelvic radiotherapy and may result in persistent neurological symptoms with a substantial impact on long-term quality of life. The primary objective of this study was to evaluate the association between radiation dose distribution to the lumbosacral plexus (LSP) and the occurrence of radiation-induced lumbosacral plexopathy (RILP).

Methods: A total of 175 cancer-free patients under long-term follow-up after pelvic radiotherapy were evaluated for lumbosacral radicular symptoms using the Oswestry Disability Index (ODI). The LSP was delineated retrospectively on planning CT scans from the L4-L5 interspace to the level of the sciatic nerve, with neuroradiologist support and pelvic MRI when available. Dosimetric parameters were extracted from treatment plans calculated using the anisotropic analytical algorithm. Correlations between dosimetric variables and clinical outcomes were analyzed using Spearman's rank correlation, with statistical significance set at p<0.05.

Results: The mean patient age was 63.3 years, with 48% female, and the median follow-up was 81.7 months. Gynecological and prostate cancers accounted for 49% and 28% of cases, respectively. Intensity-modulated radiation therapy (IMRT) was used in 35.4% of patients, and 21.1% received stereotactic pelvic radiotherapy. RILP was observed in 13% of patients, all classified as Grade 3. The median maximum dose (Dmax) to the LSP was 69 Gy, and the mean V50 Gy was 72.4%. The mean LSP volume overlapping the planning target volume was significantly higher in symptomatic patients (54.5%) compared with asymptomatic patients (18.4%). Median maximum plexus dose was 72.1 Gy in patients with neurological toxicity versus 60.2 Gy in those without toxicity (p=0.0015).

Conclusion: Higher radiation doses to the LSP, particularly Dmax and high-dose volume exposure, were significantly associated with radiation-induced lumbosacral radiculopathy. Gender and pre-existing diabetes also emerged as predictive factors. These findings support the inclusion of the LSP as an organ at risk and highlight the importance of individualized dose constraints to reduce neurological toxicity in pelvic radiotherapy.

## Introduction

Radiation-induced peripheral neuropathy is a chronic, progressive, and often irreversible complication that typically manifests several years after radiotherapy [1]. Although relatively uncommon, its incidence is rising in parallel with improvements in long-term cancer survival, leading to a growing population of patients at risk for late neurological sequelae. Lumbosacral plexopathy represents a specific form of radiation-induced neuropathy and may result in persistent motor weakness, sensory deficits, and functional impairment of the lower extremities when the lumbosacral plexus (LSP) is exposed during pelvic radiotherapy [2].

Despite the availability of extensive data and well-established dose constraints for radiation-induced brachial plexopathy, lumbosacral plexopathy remains comparatively under-recognized and insufficiently characterized [3]. This underestimation is particularly concerning given its substantial and often permanent impact on long-term quality of life, autonomy, and functional independence in cancer survivors, many of whom are otherwise disease-free. The progressive and typically irreversible nature of radiation-induced neuropathy underscores the importance of improved awareness, prevention, and risk stratification in contemporary pelvic radiotherapy [4].

The primary objective of this study was to evaluate the association between radiation dose distribution to the LSP and the occurrence of radiation-induced lumbosacral plexopathy (RILP) in patients treated for pelvic malignancies using intensity-modulated and stereotactic radiotherapy techniques. The secondary objective was to assess the prevalence of lumbosacral radicular symptoms and to explore the influence of patient-related and treatment-related factors on clinical outcomes.

## Materials and methods

Patient selection

This study included 175 patients who had been treated with radiation therapy for pelvic cancers (cervical, endometrial, prostate, and bladder) between 02 January 2017 and 02 January 2023. All patients were declared in complete remission, and they were assessed for symptomatic lumbosacral radiculopathy using the Oswestry Disability Index (ODI) [5]. Symptomatic lumbosacral plexopathy was defined as an ODI score ≥21%, corresponding to at least moderate disability. Relevant clinical and treatment data were retrieved from patients’ electronic medical records in DxCare, and follow-up assessments were conducted during oncology and radiotherapy consultations. 

Inclusion criteria

Eligible patients were adults (≥18 years) with histologically confirmed pelvic cancer and no evidence of locoregional or distant metastasis. Patients were treated using intensity-modulated radiation therapy (IMRT) techniques, had a minimum follow-up period of 24 months, and had an adequate performance status (Eastern Cooperative Oncology Group (ECOG) 0-2) [6]. All patients were able to comply with regular follow-up visits for assessment of treatment response and monitoring of treatment-related toxicities. Written informed consent was obtained from all patients after they were fully informed of the risks and benefits of treatment.

Exclusion criteria

Patients with distant metastases (M1) were excluded, as were those unable to provide informed consent; patients with a history of peripheral neuropathies unrelated to radiation; those who had undergone previous pelvic or lumbosacral surgery affecting the LSP; and patients with active infections, severe uncontrolled medical conditions, or poor performance status (ECOG >2) [6].

Lumbosacral plexus delineation

Magnetic resonance imaging (MRI)-based descriptions were primarily used when available, as CT-based information on the LSP was limited. A radiologist assisted with the identification of the LSP and adjacent structures on axial CT images. The LSP was referenced to identifiable bony and soft tissue landmarks, beginning from the L4-L5 interspace inferiorly to the level of the sciatic nerve. The referenced structures included the psoas, iliacus, piriformis, obturator internus, and gluteus maximus muscles, the common and internal iliac arteries and veins, and relevant vertebral bodies and sacral bones. The LSP is divided into three portions, referenced to the piriformis muscle: the preplexal segment (L4, L5, S1), which lies cranial to the piriformis; the plexal segment, which lies anterior to the piriformis; and the postplexal segment (sciatic nerve), which lies caudal to the piriformis and within the greater sciatic foramen [7]. Using these guidelines, the LSP was contoured retrospectively in 175 representative patients who underwent IMRT and stereotactic pelvic radiotherapy for pelvic cancer. Interobserver variability was not formally assessed; however, contouring was performed according to standardized anatomical guidelines, with neuroradiologist support to ensure consistency.

Treatment delivery

Conventional radiotherapy was delivered using two linear accelerators (Clinac IX and TrueBeam, Varian). Treatments were delivered using standard fractionation schedules of 1.8-2 Gy per fraction, administered once daily, five days per week. Image-guided radiotherapy (IGRT) was systematically implemented before each session to confirm and adjust patient positioning. Cone-beam CT (CBCT) imaging was performed during the first three treatment fractions and subsequently once per week, while daily kilovoltage (kV-kV) imaging was used for the remaining sessions to ensure consistent alignment and maintain treatment accuracy throughout the entire treatment course.

Stereotactic radiotherapy delivered by a dedicated linear accelerator (TrueBeam, Varian) was also used in patients with low-risk and favorable intermediate-risk prostate cancer [8]. Treatments were delivered according to stereotactic body radiotherapy (SBRT) protocols, using fractionation schemes commonly applied in prostate SBRT, with fractions administered every other day to optimize radiobiological efficacy and normal-tissue recovery; the selected dose and fractionation were individualized based on dose constraints to adjacent organs at risk. Image guidance was systematically performed before each session to ensure high-precision targeting, and strict immobilization procedures were applied to maintain reproducibility throughout the treatment course.

Brachytherapy was administered as a complement to external beam radiotherapy in patients with cervical and endometrial cancers [9,10]. High-dose-rate (HDR) intracavitary brachytherapy was delivered using applicators adapted to tumor anatomy, with individualized treatment planning performed on CT. Fractionation schedules followed institutional and international guidelines, and image guidance ensured accurate applicator positioning and optimal target coverage.

Data collection and statistics

Dosimetric data were extracted from the treatment plans, including dose-volume histogram (DVH) parameters describing the distribution of radiation dose within the target volume and surrounding organs at risk, including the three segments of the LSP. The biologically effective dose (BED) was calculated assuming an α/β ratio of 3 Gy, consistent with late-responding normal neural tissue. The selected dosimetric thresholds were based on dose ranges previously reported to be associated with radiation-induced peripheral neuropathy, particularly lumbosacral plexopathy, with increased risk observed for physical doses ≥50 Gy and corresponding EQD2/BED values assuming a low α/β ratio for late-responding neural tissue [7]. Associations between dosimetric parameters and clinical outcomes were assessed using Spearman's rank correlation analysis. Statistical analyses were performed using SPSS software (version 10.0) (IBM Corp., Armonk, NY, USA), with a two-sided significance threshold set at p<0.05.

## Results

The mean age of patients at the time of treatment was 63.3 years, with 48% of the patients being female. The median follow-up time was 81.7 months, 49% had gynecological cancer, while 28% had prostate cancer. Static IMRT was used in 35.4% of the cases, and stereotactic pelvic radiotherapy was applied to 21.1% of the patients. Based on ODI assessment, lumbosacral plexopathy was identified in 13% of patients, all of whom had ODI scores ≥21%, corresponding to at least moderate functional disability [5]. These patients exhibited symptoms such as pain, weakness, and sensory deficits in the lower extremities. A diagnosis of RILP was confirmed (Table 1).

**Table 1:** Clinical characteristics of the patients RILP: radiation-induced lumbosacral plexopathy; BMI: body mass index; SBRT: stereotactic body radiotherapy; IMRT: intensity-modulated radiation therapy; VMAT: volumetric modulated arc therapy

Parameter	Subcategory	All patients, n=175 (%)	Patients with symptomatic RILP, n=23
				Mean age	<60 years	84 (48%)	15
					>60 years	91 (52%)	8
Sex	Male	91 (52%)	3
	Female	84 (48%)	20
BMI	High (>25)	144 (82.2%)	17
Comorbidity	Pre-existing diabetes	43 (24.5%)	5
Tumor site	Gynecological	86 (49%)	14
	Prostatic	49 (28%)	5
	Other	40 (23%)	4
Concomitant chemotherapy	Received	56 (32%)	14
Radiotherapy technique	Static IMRT	62 (35.4%)	17
	VMAT-tomotherapy	57 (32.5%)	2
	SBRT	37 (21.1%)	3
	Brachytherapy	19 (10.8%)	1

The average maximum dose (Dmax) to the LSP was 69 Gy (±10.5 Gy). The average volume receiving 50 Gy (V50 Gy) was 72.4%. The mean volume of the LSP receiving more than 70 Gy (V70) was 6.81% in symptomatic patients versus 2.93% in asymptomatic patients.

Patients with symptomatic lumbosacral radiculopathy exhibited significantly higher high-dose exposure of the LSP. The median maximum dose to the LSP was 72.1 Gy in patients with neurological toxicity, compared with 60.2 Gy in those without toxicity. A total of 28.3% of the LSP volume was included in the planning target volume (PTV). These differences were statistically significant (p=0.0015). (Table 2).

**Table 2:** Dosimetric analysis for patients with and without RILP RILP: radiation-induced lumbosacral plexopathy; LSP: lumbosacral plexus; PTV: planning target volume

Dosimetric parameters	All patients	Patients with symptomatic RILP	Patients without symptomatic RILP
	n=175 (%)	n=23	n=152
				Mean volume (mL)	95.6	89.1	104.5
Mean LSP volume in PTV (%)	28.3	54.5	18.4
Median max dose to LSP (Gy)	69	72.1	60.2
				Mean volume receiving 50 Gy (V50) n in %	72.4	78.4	51.6
Mean volume receiving 60 Gy (V60) in %	9.2	7.4	2.4				
				Mean volume receiving more than 70 Gy (V70) in %	3.5	6.81	2.93

Beyond dosimetric parameters, several patient-related variables were also found to be significantly associated with the occurrence of RILP. Gender showed a statistically significant correlation with RILP incidence, with one group exhibiting higher rates of plexus toxicity. Similarly, concomitant chemotherapy was identified as a contributing factor, as patients receiving combined chemoradiotherapy experienced higher frequencies of RILP compared with those treated with radiotherapy alone. In addition, pre-existing diabetes mellitus emerged as an independent predictor; diabetic patients demonstrated a markedly higher risk of developing RILP. These associations suggest that clinical characteristics, alongside dose-related factors, play an important role in the likelihood of plexus injury.

## Discussion

In our study, 13% of patients exhibited clinical symptoms of lumbosacral plexopathy, all classified as Grade 3. This incidence is consistent with findings reported by other studies, which observed an incidence of 7%-20% of lumbosacral plexopathy in patients receiving pelvic radiation therapy for lower gastrointestinal cancers treated with IMRT (Table 3). However, our results differ slightly from those of other studies in the United States, which reported a lower incidence. Lumbosacral plexopathy was noted in 1.3% of patients after abdominal irradiation and in 0.32% of patients after pelvic irradiation [3]. The variation in incidence rates could be attributed to differences in patient demographics, radiation techniques, and dosimetric parameters.

**Table 3:** Literature review of radiotherapy studies on RILP RILP: radiation-induced lumbosacral plexopathy; IMRT: intensity-modulated radiation therapy

Study	Incidence of RILP	Radiation technique		Dose range	Risk factors	Key findings
Delanian et al. (2012) [11]	N/A	Radiotherapy (unspecified)	N/A		Gender, diabetes	Female gender and diabetes linked to higher RILP risk
Yi et al. (2012) [12]	7%-20%	IMRT	Doses above 50 Gy		Age, treatment site	High correlation between doses >50 Gy and RILP
Dahbi, et al. (2023) [13]	N/A	IMRT, stereotactic radiotherapy	Doses >50 Gy		Gender, diabetes	Correlation between high doses (>50 Gy) and RILP incidence in pelvic cancers
Krkoska et al. (2022) [14]	N/A	Radiotherapy for cervical cancer	Doses >50 Gy		N/A	Case of lumbosacral plexopathy with doses >50 Gy
Mottet et al. (2021) [15]	N/A	Prostate cancer radiotherapy	Doses >66 Gy		Age, gender	Doses >66 Gy associated with higher toxicity rates
Dahbi et al. (2025) (current study)	13% (Grade 3)	IMRT, stereotactic radiotherapy, and brachytherapy	Mean: 69 Gy		Gender, diabetes	Doses >70 Gy correlated with higher toxicity, median 72.1 Gy for symptomatic

Our study revealed that the average Dmax to the LSP was 69 Gy (±10.5 Gy), with higher doses (above 70 Gy) being significantly associated with neurological toxicity. These doses were administered to 67.9% of the patients treated with IMRT, which has been shown to provide better sparing of healthy tissues compared to conventional radiation techniques [12,13]. Our results demonstrate a strong dosimetric correlation, with median and mean LSP doses, as well as the mean LSP volume receiving 50 Gy, being significantly higher in patients with neurological toxicity.

Similarly, it was found that IMRT can reduce the exposure of surrounding healthy tissues through more precise radiation distribution and higher fractionated doses (1.8-2.5 Gy per cycle per day) [12-15]. Although there are no specific consensus cut-off values for RILP, a total radiation dose exceeding 50 Gy to the LSP is believed to increase the risk [16]. Additionally, a total dose of 81 Gy is significantly associated with a higher risk of genitourinary and gastrointestinal toxicity compared to a 66 Gy dose [17]. Various factors need to be considered to find the best risk-benefit balance for the patient. Long-term studies are necessary to better assess the long-term toxicity of radiation. Our findings align with their conclusion that higher radiation doses correlate strongly with the occurrence of neurological toxicity.

We also identified associations between gender and diabetes and the occurrence of RILP, with female patients and those with diabetes exhibiting a higher prevalence of neurological symptoms. Similar associations have been reported in previous studies, in which female sex and comorbid conditions such as diabetes were linked to an increased risk of radiation-induced neuropathy [11-13]. However, as multivariate analysis was not performed, these findings should be interpreted with caution and cannot be considered independent predictive factors. Rather, our results support the hypothesis that these patient-related characteristics may contribute to susceptibility to radiation toxicity and warrant further investigation in adequately powered multivariable studies.

Overall, our study's findings are consistent with existing literature on the incidence and dosimetric correlation of radiation-induced lumbosacral radiculopathy (Table 3). The identification of gender and diabetes as predictive factors aligns with other studies, highlighting the importance of considering patient-specific factors in treatment planning [12,13,17]. The use of advanced radiation techniques like IMRT appears to reduce the risk of severe toxicity, corroborating findings from other research. By comparing our results with those of previous studies, we underscore the importance of dose constraints and the need for personalized treatment approaches to minimize adverse effects and improve patient outcomes [17,18].

This study has several limitations that should be acknowledged. Its retrospective design may introduce selection and reporting biases inherent to observational analyses. The assessment of lumbosacral plexopathy relied on the ODI, which, although validated for functional disability, may not fully capture neurological specificity in the absence of standardized neurological examination or electrodiagnostic confirmation. In addition, no multivariate analysis was performed; therefore, associations observed with patient-related factors such as gender, diabetes, and concomitant chemotherapy should be interpreted as descriptive findings rather than independent predictors. Finally, the heterogeneity of tumor sites and radiotherapy techniques, while reflective of real-world practice, may confound dose-toxicity relationships and limit the generalizability of specific dosimetric thresholds.

Future directions

Further research is warranted to establish more precise dose thresholds and to explore the underlying mechanisms of radiation-induced nerve damage. Prospective studies with larger patient cohorts and advanced imaging techniques could provide deeper insights into optimizing radiation therapy while minimizing adverse effects.

## Conclusions

Radiation-induced lumbosacral radiculopathy represents a meaningful late toxicity of pelvic radiotherapy that can substantially impair long-term quality of life. Our findings demonstrate a clear association between increased radiation dose to the lumbosacral plexus and the development of neurological symptoms, emphasizing the clinical importance of careful plexus delineation and dosimetric assessment. Associations with patient-related factors such as gender and diabetes highlight the need for individualized risk awareness, although causality cannot be confirmed. While specific dose constraints cannot yet be recommended, this study provides strong clinical rationale for routinely considering the lumbosacral plexus as an organ at risk in IMRT and stereotactic pelvic radiotherapy. Future prospective studies are essential to establish validated dose thresholds and optimize neurotoxicity prevention.
